# Supplementary material for: Vulnerability and tuberculosis treatment outcomes in urban settings in England: A mixed-methods study
Source: PLoS One. 2023 Aug 17;18(8):e0281918. doi: 10.1371/journal.pone.0281918 (PMC10434856; doi:10.1371/journal.pone.0281918)
Supplement: S1 File — (DOCX) [file pone.0281918.s001.docx]

**Vulnerability and tuberculosis treatment outcomes in urban settings in England: a mixed-methods study.**

Luis C. Berrocal-Almanza et al.

**Supporting information**

**Study design**

There was no one else present besides the participant and the interviewer during the interview. No repeat interviews were carried out. Field notes were made during the interview. All interviews lasted on average one hour. The transcripts of the interviews were not returned to participants for comment and they did not provide feedback on the findings

**Data sources**

The Index of Multiple Deprivation 2015 is the official measure of relative deprivation for lower-layer super output areas, based on the 2011 census in England [1]. It ranks every small area in England from the most to least deprived. It uses deprivation deciles calculated by ranking the 32,844 small areas in England from the most to the least deprived and dividing them into 10 equal groups [1]. It is constructed by combining the following seven domains of deprivation: income, employment, education, skills and training, health and disability, crime, barriers to housing and services, and living environment [1].

For the social risk factors drug and alcohol problems, homelessness and imprisonment the registers categorized these variables as ever experienced one of this factors.

**Multiple imputation**

We used a multiple imputation by chained equations (MICE) model to impute missing values, while accounting for the uncertainty in missing information [2-4]. The creation and analysis of 20 imputed datasets is described in this section.

The percentage of missing information and the models used to impute each variable is shown in supplementary table 1. All variables used in the estimation of main effects were part of the imputation model. The predictor variables and the imputed variables were included in the model iteratively with 10 iterations per missing variable per imputation, the burn-in period was 10 and the seed set at 53421. We initially performed a complete case analysis using logistic regression models, followed by the creation and analysis of 20 imputations. The imputation model was assessed after estimation using the average relative variance increase (RVI), the largest fraction of missing information (FMI) and the Monte Carlo error (MCE) estimate. We used trace plots to assess model converge. The results of the complete case analysis did not differ from those of the data with multiple imputations.

**Supplementary tables**

**Table S1.** **Percentage of missing information, variables and models used for multiple imputation.**

| **Variable** | **Observed n (%)** | **Missing n (%)** | **Model** |
| --- | --- | --- | --- |
| Disease site | 2,245 (99.69) | 7 (0.31) | Multinomial logistic regression |
| Alcohol problems | 2,149 (95.21) | 103 (4.79) | Augmented logistic regression |
| Homelessness | 1,484 (48.3) | 768 (51.7) | Augmented logistic regression |
| Imprisonment | 1,480 (47.9) | 772 (52.1) | Augmented logistic regression |
| Drug problems | 2,119 (93.73) | 133 (6.27) | Augmented logistic regression |
| Deprivation index | 2,192 (97.27) | 60 (2.73) | Ordered logistic regression |

**Table S2 Complete case univariate analysis of factors associated with poor treatment outcome**

|  | Birmingham  OR (95% CI) | p-value | Leicester  OR (95% CI) | p-value | All participants  OR (95% CI) | p-value |
| --- | --- | --- | --- | --- | --- | --- |
| **Age (years)** |  |  |  |  |  |  |
| 16-24 | 0.9 (0.4-2.0) | 0.815 | 2.4 (1.1-5.4) | 0.026 | 1.54 (0.9-2.6) | 0.111 |
| 25-34 | Ref |  | Ref |  | Ref |  |
| 35-44 | 1.0 (0.5-1.8) | 0.946 | 1.3 (0.5-3.0) | 0.487 | 1.12 (0.6-1.8) | 0.632 |
| 45-54 | 1.7 (0.9-3.1) | 0.076 | 1.0 (0.3-2.6) | 0.100 | 1.4 (0.8-2.5) | 0.130 |
| 55-65 | 1.6 (0.8-3.1) | 0.116 | 2.5 (1.0-5.9) | 0.032 | 1.9 (1.1-3.1) | 0.011 |
| >65 | 5.2 (3.1-8.7) | <0.0001 | 7.3 (3.5-15) | <0.0001 | 5.7 (3.7-8.8) | <0.0001 |
| **Sex** |  |  |  |  |  |  |
| Female | Ref |  | Ref |  | Ref |  |
| Male | 1.1 (0.8-1.6) | 0.310 | 1.1 (0.7-1.7) | 0.556 | 1.1 (0.8-1.5) | 0.264 |
| **World region of origin** |  |  |  |  |  |  |
| Americas | 2.4 (0.8-7.0) | 0.087 | -- | -- | 2.5 (0.9-6.9) | 0.077 |
| Central Europe | 2.6 (1.2-5.8) | 0.013 | 13 (3.8-47) | <0.0001 | 3.9 (2.0-7.3) | <0.0001 |
| East Asia | 2.4 (0.5-12) | 0.261 | 11 (1.6-81) | 0.013 | 3.8 (1.1-12) | 0.026 |
| East Europe | 2.4 (0.2-22) | 0.421 | 8.7 (0.7-108) | 0.092 | 3.5 (0.6-17) | 0.130 |
| East Mediterranean | 1.9 (0.4-9.4) | 0.387 | 17 (0.9-308) | 0.051 | 2.8 (0.7-10) | 1.114 |
| North Africa | -- | -- | -- | -- | -- | -- |
| South Asia | 1.2 (0.7-1.8) | 0.369 | 1.4 (0.6-3.2) | 0.426 | 1.1 (0.7-1.6) | 0.532 |
| South East Asia | 1.6 (0.4-5.9) | 0.437 | -- | -- | 0.9 (0.2-3.1) | 0.909 |
| Sub Saharan Africa | 1.0 (0.6-1.9) | 0.802 | 2.5 (0.9-6.6) | 0.051 | 1.3 (0.8-2.1) | 0.205 |
| United Kingdom | Ref |  | Ref |  | Ref |  |
| West Europe | 1.1 (0.3-3.8) | 0.875 | 5.8 (1.2-26) | 0.023 | 1.8 (0.7-4.6) | 0.183 |
| **Deprivation index** |  |  |  |  |  |  |
| 1-2 decile (most deprived) | Ref |  | Ref |  | Ref |  |
| 3-4 decile | 1.2 (0.7-2.0) | 0.349 | 0.8 (0.4-1.3) | 0.379 | 1.1 (0.8-1.5) | 0.459 |
| 5-6 decile | 0.9 (0.4-1.7) | 0.763 | 0.3 (0.1-0.9) | 0.036 | 0.6 (0.3-1.1) | 0.166 |
| 7-8 decile | 1.4 (0.6-3.0) | 0.383 | 0.3 (0.09-1.0) | 0.066 | 0.8 (0.4-1.6) | 0.625 |
| 9-10 decile (least deprived) | 0.7 (0.2-2.6) | 0.692 | 0.7 (0.2-1.9) | 0.502 | 0.8 (0.4-1.8) | 0.713 |
| **Drug problems** |  |  |  |  |  |  |
| Yes | 1.4 (0.7-2.8) | 0.328 | -- | -- | 1.3 (0.6-2.6) | 0.378 |
| No | Ref |  |  |  | Ref |  |
| **Homelessness** |  |  |  |  |  |  |
| Yes | 1.1 (0.3-3.2) | 0.812 | -- | -- | 1.1 (0.3-3.2) | 0.812 |
| No | Ref |  |  |  | Ref |  |
| **Imprisonment** |  |  |  |  |  |  |
| Yes | 1.3 (0.6-3.0) | 0.462 | -- | -- | 0.7 (0.3-1.6) | 0.462 |
| No | Ref |  | Ref |  | Ref |  |
| **Alcohol problems** |  |  |  |  |  |  |
| Yes | 1.9 (0.7-5.1) | 0.194 | 2.2 (0.8-6.0) | 0.118 | 1.8 (0.9-3.8) | 0.071 |
| No | Ref |  | Ref |  | Ref |  |
| **Disease site** |  |  |  |  |  |  |
| Pulmonary | Ref |  | Ref |  | Ref |  |
| Extra-pulmonary | 0.8 (0.5-1.2) | 0.369 | 0.5 (0.3-0.8) | 0.006 | 0.8 (0.5-1.3) | 0.624 |
| Both | 1.1 (0.6-2.0) | 0.604 | 0.4 (0.2-0.8) | 0.017 | 1.2 (0.8-1.8) | 0.349 |
| **Smear test** |  |  |  |  |  |  |
| Negative | Ref |  | Ref |  | Ref |  |
| Positive | 0.7 (0.4-1.2) |  | 1.3 (0.7-2.3) |  | 0.9 (0.6-1.3) | 0.791 |
| **MDR-TB** |  |  |  |  |  |  |
| Yes | 3.8 (1.4-10) | 0.007 | -- | -- | 3.2 (1.2-8.5) | 0.015 |
| No | Ref |  |  |  | Ref |  |

**Table S3:** **Complete case multivariate analysis of factors associated with poor treatment outcome.**

|  | All participants  OR (95% CI)  n=1,417 | p-value | Birmingham  OR (95% CI)  n=1,417 | p-value | Leicester  OR (95% CI)  n=595 | p-value |
| --- | --- | --- | --- | --- | --- | --- |
| **Age (years)** |  |  |  |  |  |  |
| 16-24 | 1.20 (0.51-2.84) | 0.668 | 1.20 (0.51-2.84) | 0.668 | 2.82 (0.77-10.3) | 0.115 |
| 25-34 | Ref |  | Ref |  | Ref |  |
| 35-44 | 1.13 (0.56-2.27) | 0.718 | 1.13 (0.56-2.27) | 0.718 | 1.85 (0.52-6.54) | 0.338 |
| 45-54 | 1.80 (0.90-3.61) | 0.093 | 1.80 (0.90-3.61) | 0.093 | 1.40 (0.29-6.78) | 0.671 |
| 55-65 | 2.39 (1.18-4.80) | 0.014 | 2.39 (1.18-4.80) | 0.014 | 1.09 (0.21-5.57) | 0.917 |
| >65 | 8.63 (4.65-16.0) | <0.0001 | 8.63 (4.65-16.0) | <0.0001 | 5.66 (1.59-20.1) | 0.007 |
| **Sex** |  |  |  |  |  |  |
| Female | Ref |  | Ref |  | Ref |  |
| Male | 1.37 (0.93-2.02) | 0.106 | 1.37 (0.93-2.02) | 0.106 | 1.06 (0.50-2.23) | 0.869 |
| **World region of origin** |  |  |  |  |  |  |
| Americas | 1.85 (0.60-5.71) | 0.283 | 1.85 (0.60-5.71) | 0.283 | --- | --- |
| Central Europe | 4.81 (2.02-11.4) | <0.0001 | 4.81 (2.02-11.4) | <0.0001 | 34.1 (3.76-310) | 0.002 |
| East Asia | 0.92 (0.10-8.40) | 0.947 | 0.92 (0.10-8.40) | 0.947 | 53.4 (1.60-178) | 0.026 |
| East Europe | 3.50 (0.33-36.4) | 0.293 | 3.50 (0.33-36.4) | 0.293 | 17.01 (0.84-343) | 0.065 |
| East Mediterranean | 2.41 (0.45-12.8) | 0.300 | 2.41 (0.45-12.8) | 0.300 | --- | --- |
| North Africa | --- | --- | --- | --- | --- | --- |
| South Asia | 1.06 (0.64-1.75) | 0.800 | 1.06 (0.64-1.75) | 0.800 | 1.63 (0.34-7.77) | 0.537 |
| South East Asia | 1.53 (0.37-6.28) | 0.549 | 1.53 (0.37-6.28) | 0.549 | --- | --- |
| Sub Saharan Africa | 1.40 (0.72-2.69) | 0.315 | 1.40 (0.72-2.69) | 0.315 | 3.47 (0.68-17.6) | 0.134 |
| United Kingdom | Ref |  | Ref |  | Ref |  |
| West Europe | 0.72 (0.19-2.74) | 0.638 | 0.72 (0.19-2.74) | 0.638 | 6.42 (0.78-52.6) | 0.083 |
| **Deprivation index** |  |  |  |  |  |  |
| 1-2 decile (most deprived) | Ref |  | Ref |  | Ref |  |
| 3-4 decile | 1.04 (0.60-1.80) | 0.881 | 1.04 (0.60-1.80) | 0.881 | 0.67 (0.30-1.50) | 0.335 |
| 5-6 decile | 0.79 (0.39-1.61) | 0.530 | 0.79 (0.39-1.61) | 0.530 | 0.17 (0.02-1.38) | 0.099 |
| 7-8 decile | 1.36 (0.59-3.16) | 0.467 | 1.36 (0.59-3.16) | 0.467 | 0.14 (0.01-1.47) | 0.102 |
| 9-10 decile (least deprived) | 0.69 (0.19-2.46) | 0.575 | 0.69 (0.19-2.46) | 0.575 | 0.40 (0.04-3.45) | 0.412 |
| **Drug problems** |  |  |  |  |  |  |
| Yes | 1.58 (0.57-4.35) | 0.371 | 1.58 (0.57-4.35) | 0.371 | -- | -- |
| **Homelessness** |  |  |  |  |  |  |
| Yes | 1.15 (0.27-4.91) | 0.845 | 1.15 (0.27-4.91) | 0.845 | -- | -- |
| **Imprisonment** |  |  |  |  |  |  |
| Yes | 0.79 (0.27-2.31) | 0.674 | 0.79 (0.27-2.31) | 0.674 | -- | -- |
| **Alcohol problems** |  |  |  |  |  |  |
| Yes | 0.48 (0.14-1.64) | 0.247 | 0.48 (0.14-1.64) | 0.247 | 1.67 (0.44-6.22) | 0.444 |
| **Disease site** |  |  |  |  |  |  |
| Pulmonary | Ref |  | Ref |  | Ref |  |
| Extra-pulmonary | 1.22 (0.81-1.84) | 0.326 | 1.22 (0.81-1.84) | 0.326 | 0.43 (0.18-1.00) | 0.051 |
| Both | 1.51 (0.82-2.80) | 0.184 | 1.51 (0.82-2.80) | 0.184 | 0.49 (0.18-1.30) | 0.156 |
| **MDR-TB** |  |  |  |  |  |  |
| Yes | 5.31 (1.79-15.7) | 0.003 | 5.31 (1.79-15.7) | 0.003 | -- | -- |

**References**

1. Ministry of Housing, Communities & Local Government. English indices of deprivation 2015 2015 [Available from: <https://www.gov.uk/government/statistics/english-indices-of-deprivation-2015>.

2. StataCorp. Multiple-imputation reference manual. Stata: Release 15.

Statistical Software. College Station, TX: StataCorp LLC2017.

3. He YL. Missing Data Analysis Using Multiple Imputation Getting to the Heart of the Matter. Circulation-Cardiovascular Quality and Outcomes. 2010;3(1):98-U145.

4. Schafer JL. Multiple imputation: a primer. Stat Methods Med Res. 1999;8(1):3-15.
