# Supplementary material for: Vulnerability and tuberculosis treatment outcomes in urban settings in England: A mixed-methods study
Source: PLoS One. 2023 Aug 17;18(8):e0281918. doi: 10.1371/journal.pone.0281918 (PMC10434856; doi:10.1371/journal.pone.0281918)
Supplement: Attachment — Submitted filename: review.docx [file pone.0281918.s002.docx]

Thank you for the opportunity to review this manuscript, which used mixed-methods to investigate risk factors for adverse treatment outcomes among people with TB in Birmingham and Leicester in England.

I really enjoyed reading this study and congratulate the authors on making use of data collected as part of routine TB registers and the ETS system to analyse risk factors for adverse treatment outcomes in a large cohort. It was also refreshing and useful to read the qualitative findings of the report, which help to add crucial insight and context. The recognition of the importance of using local data to design specific interventions is extremely welcome and relevant, as is the attention given to addressing the social and economic determinants of TB through a multi-sector response including better integration of health, social care, and social protection services. I have minimal experience of qualitative methodology and analysis and so my review therefore focusses more on the quantitative aspects.

**General points**

- Suggest review language throughout to use non-stigmatising language where possible (<https://www.stoptb.org/words-matter-language-guide>), and avoid terms such as alcohol abuse.

**Specific points**

Introduction

- It would be helpful if the authors could include some data on the local epidemiology of TB in Birmingham and Leicester.

Methods/results

- It would be helpful to include more detail in the main text on what the qualitative interviews explored and how/why the questions were developed as they were. The topic guides that have been included helpfully in the appendix should be signposted to so that people know they are there (I didn’t until I checked the supplement). However, I would be tempted to put all the additional information currently in the supplement (except for table S4) in the main text (table S3 can be usefully combined with table 1; and the additional text in the supplement is brief and could be shortened a bit).
- It would be interesting to see the breakdown of adverse outcomes (i.e. % lost to follow-up versus % stopping treatment versus % death). Did the authors consider investigating risk factors for these outcomes separately? Similarly, some more information on how these outcomes are defined and what they mean would be helpful (e.g. does death refer to death during treatment from any cause or specifically from TB?).
- It looks like the analysis was restricted to adults aged 16 and over? A sentence in the methods stating this would be helpful.
- Although table 1 presents the demographic characteristics, it would be helpful in the text to highlight some of the more striking ones (e.g. 59% male, 62% of patients living in the most deprived 20% of areas of the country)
- For each of the variables in table 1, it would be helpful to include the % of data that were missing. It wasn’t clear if the percentages were calculated using the total as the denominator or the total with data available?
- It would be helpful if more information on variable definitions was included in the main text, e.g. the fact that deprivation is based on the IMD. How were drug and alcohol use defined (current/past etc)? Did imprisonment refer to active incarceration or ever been incarcerated? Readers will want to understand more clearly how these data were collected and what they represent to be able to make judgements about their validity, and many will not be familiar with the UK systems used to collect data on TB notifications.
- The authors have presented an overall multivariable regression model. I think it would be useful also see the results of a univariable unadjusted regression analysis. I also wonder if the tables could be re-organised so that readers can study the absolute proportions with adverse outcomes alongside the ORs for each risk factor (potentially this could be in a new table alongside the unadjusted analysis).
- I wonder if the IMD should be analysed differently. Deprivation is such a strong risk factor for TB and adverse outcomes in other settings (and indeed the ORs presented are suggestive here of an association, especially in Leicester) and I wonder if the small number of people in the least deprived deciles is masking a true effect. I would be interested to see an analysis of a dichotomised variable (e.g. IMD 1-5 versus 6-10).
- Similarly, I wondered if the authors considered an analysis of any social risk factor versus none; or 2 social risk factors versus one versus none (as presented in the UKHSA TB in England report).
- The study took place over many years and care provided to patients may have changed during that time. Was year considered in the analysis as a potential predictor of bad outcome?
- Have the authors considered using population attributable fractions as a way of illustrating the public health importance of these risk factors? This might complement the current analysis nicely.

Discussion

- I think more needs to be included on the limitations of this study, particularly in terms of the quantitative data. For example, the data on social risk factors are potentially subject to significant social desirability bias and thus underreporting. I also personally didn’t completely agree with the way these results were framed as showing no association between social risk factors and adverse outcomes. The numbers of people reporting these risk factors were small, and, because of this, the data presented don’t really provide evidence showing no association between these variables and adverse treatment outcomes as the confidence intervals are so wide. Indeed, for alcohol for example, the OR point estimate is 1.8 and the confidence interval 0.6-4.9. This relates to the point above about PAFs; something can be an important individual risk factor but have much less importance at a population level because of the low population prevalence of the risk factor. It seems more likely and plausible to me that there is an association between alcohol misuse and adverse outcome that would have been detected if the study was larger, but it’s PAF is likely to be very low. I’d be interested to see more discussion around this issue in the manuscript.
- I think it’s also worth mentioning that some of the most vulnerable people (who may well have some of these social risk factors) are the people who are most likely to not have had their TB diagnosed and thus been included in this study.
- Furthermore, I think it would be worth highlighting some other variables that have been shown to be important in other settings for predicting adverse outcomes, for which data were not available/not analysed in this study (e.g. were data on smoking, smear grade, symptom duration before diagnosis, co-morbidities available?).
- I think some mention of the definition of adverse outcome not encapsulating all adverse impacts of TB would be worthwhile (e.g. some of those who completed treatment will have TB recurrence, others will have significant post-TB lung disease…and it’s plausible that social risk factors are particularly important for some of those impacts).
- The point about South Asian people having better adaptation to the UK health system is interesting. Do the authors have any data on length of time in the UK available for their participants? Is there any evidence to support that hypothesis?
- Some comparison of overall treatment success rates to the UK average would be interesting.
- Overall the discussion is very good and brings together the different aspects of the manuscript nicely to provide recommendations for action.
