# Supplementary material for: Vulnerability and tuberculosis treatment outcomes in urban settings in England: A mixed-methods study
Source: PLoS One. 2023 Aug 17;18(8):e0281918. doi: 10.1371/journal.pone.0281918 (PMC10434856; doi:10.1371/journal.pone.0281918)
Supplement: Attachment — Submitted filename: Response to reviewers.docx [file pone.0281918.s004.docx]

**Second revision point-by-point response**

6. Review Comments to the Author

Please use the space provided to explain your answers to the questions above. You may also include additional comments for the author, including concerns about dual publication, research ethics, or publication ethics. (Please upload your review as an attachment if it exceeds 20,000 characters)

Reviewer #1: Thank you for responding to many of the points raised regarding your article. I still feel some points have not been fully addressed.

**Second revision points:**
**Point 1.** The quantitative part of this study is replicating research originally conducted
on a London cohort in different settings. Further qualitative research has been
conducted but through purposive sampling of patients experiencing diagnostic delay in
addition to experiencing poor TB treatment outcomes. However, diagnostic delay was
not commented on in the quantitative analysis and these to read as different pieces of
work.
Reply: The reviewer is right that this study replicates some work done in London,
however, few studies have been performed outside of London addressing this topic, as
a result, the epidemiology of TB in London has shaped the national TB control agenda
in England. On the other hand, the main focus of this study was not to understand the
phenomenon of diagnostic delay but to get insights into healthcare priorities, service
improvement and novel interventions that might be most appropriate for the target
group taking into account its setting specific characteristics. Diagnostic delay was used
as a variable to select participants for the interviews because according to the
epidemiology in England underserved populations are mainly affected by diagnostic
delay. The reviewer is right that there was not enough mention or clarification on
diagnostic delay in the first version of the manuscript. In the revised version we
introduced diagnostic delay early on in the introduction in line 104

*Further reply:* Thank you for clarifying perhaps states in abstract p3 48-49-“urban area of England other than London”
Also I think you should clarify diagnostic delay in the abstract if possible.

**Reply:** We added to the abstract “urban areas of England other than London” in line 49 of the abstract as recommended. Regarding diagnostic delay, we could not find a way to clarify this without making the abstract too long. A midpoint solution we propose is to highlight in the abstract that the interviewed participants were purposely selected. We included this in line 55-46 of the abstract.

**Point 8:** P12 Line 126-127 This seems a bit odd as you have discussed the
requirement of locally relevant needs assessments but then are intending to combine
findings from two different cities without any further reflection on whether these
combined findings will remain locally relevant.
I also think it needs to be clear why you are defining healthcare priorities using
qualitative methodology now. Would it not make sense to determine who is likely to
have a poor treatment outcome first (from your quantitative work) – then purposively
sample those individuals you have identified as being at higher risk of poor treatment
outcomes.

Reply: We do not agree with the reviewer that the results are combined. Tables 1-3
present results for the two cities independently and then in a column representing the
full study cohort from the two cities. Thus, the reader can view both sets of results.
Likewise, in the text, the results for the two cities are presented and we highlight that
they were similar, lines 214-217. We did not find any difference that merit an
independent presentation or discussion. The same applies to the qualitative results,
the results were similar, had we found any difference between the two cities we would
have presented it in the results, but it was not the case.

*Further reply:* If this is the case perhaps a sentence to state there were no significant differences in findings between Leceister and Birmingham would be sufficient but just eyeballing there seems to be a much greater percentage of patients in Birmingham in IMD decile 1-2 than Leciester 73.6% versus 37.7%. Also Leicester cohort had no homeless or imprisoned patients included.

**Reply:** In line 266-267 of the results section we state that the results were similar in both cities **“**the multivariate analysis with poor treatment outcome demonstrated that in both settings” the reason we did not comment on the possible difference at baseline in IMD is because it did not have any effect in the primary outcome of the quantitative analysis which was our focus.

Regarding the use of qualitative methods to define healthcare priorities. This is simply
something that cannot be done using only quantitative methods. In lines 121-123 of the
introduction, we mention that “Most studies on TB treatment outcomes in underserved
populations in England have been done in London, however this may not be
representative of the national picture” For this reason, in terms of TB control, the
epidemiology of TB in London has driven control policy in England based mainly on a
few quantitative studies, and this is exactly what this study aims to address. We also
mentioned in lines 114-116 of the introduction that “intervention appropriateness
should be informed by a local needs assessment. For this, the target population must
be first defined and characterised to enable the identification of their healthcare
priorities.”

*Further Reply:* I think there is a bit of confusion here. I am not suggesting that you define the healthcare priorities using quantitative methods but that purely you could have used it to facilitate purpose sampling.

**Reply:** We agree with the reviewer that the quantitative analysis could have been used to inform purpose sampling, and when we were conceiving the study we thought to do it this way, but it was very difficult to operationalise, and our stakeholders from the National Health Service and the UK Health Security Agency needed to gather this evidence for decision making. For this reason, we opted for this approach which allowed us to gather information more readily.

On the selection of participants for the qualitative study, we think that both approaches
are valid, the one the reviewer proposed and the one we used. We selected patients
based on variables known to be related to poor treatment outcome and diagnostic
delay. The process was guided by the local lead TB nurses who were very familiar with
their patient populations. In fact, had we followed the approach suggested by the
reviewer we would most probably have ended up interviewing the same patients
because we were constrained by patients willingness to be interviewed, this was a
major challenge. According to reviewer’s comment we added a mention of this
limitation in the discussion section lines 508-510

*Further reply:* Yes agreed patients willingness to be interviewed is certainly challenging and a constraint that has to be worked within. I do think that you need to recognise that there are biases to the sampling strategy led by a TB nurse given their specific role in this context. This is part of the COREQ guidance.

**Reply:** We added a new statement mentioning this aspect as a limitation in lines 525-527.

METHODS –
**Point 9:** As mentioned above I am not sure what the justification is for performing this
work concurrently and why this has been done and what this adds.
Reply: Our answer to point 8 address this point.

*Further reply:* This has not been justified yet. Just a short statement as to what is the advantage of performing this concurrently would do? such as - Did you gain data in realtime to inform your topic guides and subsequently shape your interviews?

**Reply:** A statement on this is already in the article lines 519-525 “Capturing or assessing social vulnerability is difficult to do, and we were constrained by the approach used by the UKHSA of measuring only certain SRFs. Vulnerability is not explained by one social or demographic factor alone; it is influenced by multiple and multidimensional factors. We attempted to overcome this limitation by integrating qualitative and quantitative data to comprehend patients’ and service providers’ perspectives on the hurdles for getting a diagnosis and completing treatment”

**Point 16:** Table 1 – this details that 40% of the total patients had extrapulmonary TB –
at no point has there been any discussion about diagnosis of pulmonary versus
extrapulmonary and clinical complexity. This is vital if you are going to discuss and
select patients based on diagnostic delay. I appreciate this doesn’t appear to be what
this article set out to look at and that is why is seems somewhat confused.
Reply: We addressed the points related to diagnostic delay previously in points 1 and
10.

We think that a discussion on the diagnosis of pulmonary versus extrapulmonary
TB is out of the scope of this study.

*Further reply:* If you are going to touch on diagnostic delay in the context of TB presenting to a primary care setting. You need to put in a sentence that recognises that there is diagnostic complexity here. Especially when you are including extrapulmonary TB in your data set. If you feel it is outside of the scope of this article then I would remove the extra-pulmonary data.

**Reply:** We agree with the reviewer, we highlighted this limitation in lines 518-519.

**Point 20:** P19 – line 290-292 is it realistic/ sensible to expect TB (in a low incidence
setting) to be an immediate diagnosis that a GP considers when a patient attends with
symptoms (the majority of patients attend GP surgeries with symptoms). You should at
a minimum state – symptoms consistent with a diagnosis of TB. Furthermore,
recognise that these can be non-specific at initial presentation and that there can be
clinical complexity. It is also not clear how this relates to your primary line of
questioning for this paper. The sample here is biased as you stated in your methods
that you were selecting patients based on having had a diagnostic delay and I think
you need to justify why this was done.

Reply: The study was done in high incidence areas in Birmingham and Leicester, like
in some boroughs of London, the incidence of TB is very high in some areas of these
two cities. For this reason, the goals of the collaborative Collaborative Tuberculosis
Strategy for England 2015–2020 included to improve access to services and ensure
early diagnosis and to ensure an appropriate workforce to deliver TB control, further
information can be accessed in reference 5. Within this context, the NHS and the
UKHSA started a programme to train GPs in these areas to recognise and diagnose
TB. Thus, we think it is pertinent and relevant that TB patients suggest that GPs in
these areas should be able to recognise and diagnose TB. The results of this study
have already informed the strengthening of training for GP in high incidence areas of
Birmingham and Leicester led by the NHS and the UKHSA.

*Further reply:* Can I please clarify are you suggesting that a patient presenting with any symptom (not symptoms consistent with TB) –low mood, vaginal bleeding, infected CS scar, traumatic injuries, toothache, haemorrhoids, eczema etc etc – the list can continue. Should immediately be considered for a diagnosis of TB? I do agree with you we need to think about this more and in high incidence settings, but I think stating symptoms consistent with TB would be a much more sensible considered approach. I think you need to change the wording of page 37 line 348-350

**Reply:** We agree with the reviewer, a new sentence was added in lines 348-349 as recommended.

Furthermore, are the GP surgeries you are referring to in these data practicing in the areas of high incidence you are referring to? Are you able to back this up with your data?

**Reply:** We did not have data on GP registration for the patients that were interviewed, however we do know that they resided in high incidence areas and normally the GP practice is close to the place of residence.

4.DISCUSSION –
**Point 22:** Birmingham and Leicester are two different settings however findings have
been amalgamated and no consideration has been given to their differences in terms
of interpretation of results. You have in your introduction highlighted the importance of
local relevant data so this seems at odds with what you have done.

Reply: Although Birmingham and Leicester are different settings, they share similar
characteristics in terms of TB epidemiology and demographic make-up with large
communities with heritage from the Indian subcontinent and TB concentrated in non-
UK born individuals. We included a brief mention about this in the revised version line
127 of the introduction and cited reference 1. Likewise, we highlight in the results lines
261-263 that the qualitative results in both settings were similar and for that reason
they’re not presented separately.

*Further reply:* As stated above there are differences in percentages of homeless and imprisoned patients between the two cities and these are underserved groups you are highlighting in your introduction as important.

**Reply:** As we stated in point 8, any baseline difference did not affect the primary outcome. We already mentioned in the limitations in lines 505-506 that the TB register in Leicester did not collect information on homelessness or imprisonment.

**Point 26:** The reported qualitative findings don’t meet Consolidated criteria for reporting
qualitative research (COREQ) reporting guidelines.
https://academic.oup.com/intqhc/article/19/6/349/1791966

Reply: The items that we forgot to mention and that the reviewer spotted were
introduced in the revised

*Further reply:* The reporting doesn’t currently meet all COREQ reporting guidance please see checklist.
https://cdn.elsevier.com/promis_misc/ISSM_COREQ_Checklist.pdf

**Reply:** We added the missing information on the COREQ criteria to the supporting information and highlighted it in the main text lines 216-218
